# Mechanism of wax-ripening wheat seeds attracting birds based on GC-MS widely targeted metabolomics and electronic nose technology

**DOI:** 10.3389/fpls.2026.1805496

**Published:** 2026-04-24

**Authors:** Siyi Wang, Xiaomin Zhang, Bing Han, Yanping Xing, Dongsheng Chen, Yan Yang

**Affiliations:** 1 Inner Mongolia Agricultural University/Inner Mongolia Agricultural University, Key Laboratory of Germplasm Innovation and Utilization of Triticeae Crops at Universities of Inner Mongolia Autonomous Region, Hohhot, Inner Mongolia, China; 2 Ningxia Academy of Agricultural and Forestry Sciences, Institute of Crop Science, Yinchuan, China

**Keywords:** aromatic compounds, avian feeding preference, electronic nose detection, metabolomics analysis, sulfide compounds

## Abstract

Based on observation that house sparrows (*Passer domesticus*) and wild rock pigeons (*Columba livia*) exhibited distinct feeding preference during the wax-ripening stage showing strong attraction to the wheat cultivar Nongpin 5 and its F_1_seeds derived from the direct cross population (♀Nongpin 5×♂Mengjian 33), while actively avoiding Mengjian 33 and F_1_seeds derived from the direct cross population (♀Mengjian 33×♂Nongpin 5). Subsequently, combined analyses of metabolomics profiling and electronic nose (E-nose) detection were performed on F__1__generation seeds of two reciprocal crosses populations. The results demonstrated that the direct cross population exhibited significantly higher levels of flavor compounds linked to woody, lipidic, and fruity sensory profiles, including n-decanol and 2-phenylethanol. In contrast, the reciprocal cross population accumulated significantly higher levels of sulfides and aromatic organosulfur compounds, among which, 2-methyl-1-butanethiol identified as potential key metabolites responsible for avian food avoidance. To validate these findings, feeding experiments with domesticated pigeons were conducted using diets treated with 2-methyl-1-butanethiol, n-decanol and furanone, respectively. The results confirmed that domesticated pigeons exhibited diets supplemented with n-decanol, while actively avoiding those treated with 2-methyl-1-butanethiol. Collectively, these findings demonstrate that the differences in flavor-related metabolites among wheat grain cultivars underlies avian foraging preferences. This study also provides a solid theoretical basis for future research on wheat grain flavor modulation and the development of breeding strategies aimed at tailoring flavor profiles to mitigate avian pest pressure

## Introduction

Wheat production often suffers severe losses due to foraging by avian species such as house sparrows. Statistical data indicate that avian infestation can cause a 5-15% reduction in wheat yield, and this figure may even reach up to 30% in some regions (Tracey et al., 2015). Although traditional bird repellent methods (e.g., acoustic deterrence, chemical repellents) exhibit certain effectiveness, they are plagued by several limitations, including high costs, poor environmental adaptability, and the rapid development of avian tolerance. Therefore, the development of eco-friendly strategies based on the inherent resistance of plants is of paramount importance.

Plant volatile organic compounds (VOCs) are crucial chemical mediators in plant-environment interactions, playing multiple pivotal roles such as defending against herbivores, attracting natural enemies, and modulating microbial communities (Pare and Tumlinson, 1999). Wheat can release a variety of volatile metabolites, with over a hundred metabolites identified in current research. Based on their chemical structures and biosynthetic pathways, these compounds are mainly classified into terpenoids, fatty acid derivatives, aromatic compounds, and sulfur-containing compounds (Zhao et al., 2025). These volatile metabolites may regulate the foraging preferences of insects or avian species by releasing chemical signals of varying concentrations or by modulating the temporal dynamics of signal emission during different growth stages (Turlings and Erb, 2018).

The characterization of volatile metabolites is fundamental to understanding the flavor profiles of cereal crops. Extending this approach specifically to wheat, a comprehensive study by Tang, P., Wang, L., et al. employed headspace solid-phase microextraction coupled with gas chromatography-mass spectrometry/olfactometry (HS-SPME-GC-MS/O) to dissect the aroma composition of premium wheat varieties with distinct sensory types (e.g., “fresh,” “sweet,” and “roasty” aroma).This targeted volatilomics strategy identified dozens of key active aroma compounds, primarily including aldehydes, alcohols, ketones, pyrazines, and sulfur-containing compounds. Critically, the application of odor activity value (OAV) calculation revealed the distinct contribution of specific volatiles: hexanal and other aldehydes were dominant in the “fresh” type, while compounds like 2,3-butanedione characterized the “sweet” type, and pyrazines were pivotal for the “roasty” aroma. By systematically correlating sensory evaluation data with quantitative chemical profiles, this study conclusively demonstrates, from a quantitative perspective, that the distinctions in wheat flavor types are predominantly determined by the specific composition and concentration of their key aroma-active metabolites, thereby providing a robust chemical foundation for the perceived sensory attributes (Tang et al., 2024).

Studies focusing on the spatiotemporal distribution of volatile metabolites in cereal crops have yielded valuable insights. From a developmental perspective, the variety and content of volatile compounds in wheat are significantly higher during the grain filling stage than in other growth periods, which is likely closely associated with the enhanced metabolic activity during grain development (Yang et al., 2020). In terms of tissue distribution, high levels of terpenoids are also emitted from various plant tissues, including spikes, leaves, and stems. Relevant studies on wheat have further confirmed that the emission of terpenoids may serve as one of the essential conditions for attracting the natural enemies of aphids (Bruce et al., 2015).

During avian foraging, volatile compounds may act as “chemical signals” to mediate interspecific interactions. For example, studies on intraspecific and interspecific relationships mediated by plant volatiles have suggested that the emission of terpenoids may attract seed-eating birds and herbivorous insects, while certain aldehydes may be avoided by birds and herbivorous insects due to their pungent odors (Kohl et al., 2015). Existing studies have shown that blackbirds exhibit a distinct feeding preference for fruits containing lipid compounds such as β-pinene, and house sparrows are sensitive to changes in the concentration of hexanal in wheat grains (Stiles, 1993). In a previous genome-wide association study (GWAS), a key gene regulating avian feeding behavior in sorghum, *Tannin1* (encoding a WD40 protein), was identified. This study revealed that functionally intact Tannin1 promotes the accumulation of anthocyanins and proanthocyanidins (PAs) in terms of defensive metabolite synthesis, which significantly reduces the feeding preference of house sparrows. Regarding the production of attractive volatiles, mutant *tan1-a/b* alleles lead to the downregulation of SbGL2 expression, resulting in higher levels of C6-C9 volatile aldehydes (e.g., hexanal and 1-octen-3-ol) via the fatty acid oxidation pathway, which increases the residence time of house sparrows by 40% (Xie et al., 2019).

Therefore, investigating the intrinsic volatile metabolites of wheat and analyzing the effects of their types or contents on avian feeding preferences is a crucial subject for reducing losses in wheat production.

During the maturation stage in wheat germplasm nurseries, a long-term observation revealed significant intervarietal differences in the feeding preferences of house sparrows and wild rock pigeons for wheat grains. These avian species exhibit distinct feeding preferences for specific wheat cultivars. Similarly, they display significant differences in feeding preference to F_1_ seeds between the direct cross population and reciprocal populations, derived from the same two parental cultivars. Based on these observations, this study utilized mixed pools of wax-ripened F_1_seeds from both reciprocal crosses F_1_population (Nongpin 5×Mengjian 33) as research materials. High-throughput metabolomics and electronic nose (E-nose) detection were employed to qualitatively and quantitatively analyze the primary and secondary metabolites and screen key volatile compounds that may affect the potential feeding preference and avoidance of birds, and to clarify the underlying mechanisms of avian feeding preference for wheat seeds.

## 2 Materials and methods

### 2.1 Materials preparation

In 2024, direct and reciprocal crossing experiments between cultivars Nongpin 5 and Mengjian 33 were conducted in the wheat germplasm nursery at the Science and Technology Park of Inner Mongolia Agricultural University. The flowers were bagged to prevent outcrossing, and F_0_hybrid seeds were harvested. In 2025, F_1_seeds at the wax-ripening stage from both the direct and reciprocal crosses of Nongpin 5 × Mengjian 33 were obtained and stored at -80°C.

Mature seeds of cultivar Yongliang 4 was used to pigeon feeding experiments.

### 2.2 Sample preparation

For targeted metabolomic analysis and E-nose detection, mixed samples were prepared separately from the waxy-ripe grains of the direct cross F_1_generation (bird-preferred type) derived from ♀Nongpin 5×♂Mengjian 33, and the waxy-ripe grains of the reciprocal cross F_1_generation (bird-avoided type) derived from ♀Mengjian 33×♂Nongpin 5. Three biological replicates were set for each sample to ensure the reliability of the experimental results.

### 2.3 E-nose detection of flavor compounds in wheat grains

Exactly 2.0 g of each sample was weighed into a 15 mL headspace vial, which was then sealed and equilibrated at room temperature for 30 minutes. The analysis was performed using an AIRSENSE PEN3 E-nose with the following parameter settings: sensor self-cleaning time and zeroing time were 120 seconds and 5 seconds, respectively; injection flow rate was 300 mL/min; and data collection time was 120 seconds. The experiment collected the overall odor information of samples through an array of 10 metal oxide sensors (**Table 1**) (e.g., W1W is sensitive to sulfides, W5S is sensitive to nitrogen oxides). Principal component analysis (PCA) and loadings analysis were applied to process the sensor response data to visualize and quantify the odor differences between different samples and identify the key discriminant compounds.

**Table 1:** Response data of electronic nose sensors.

Serial no.	Sensor name	Performance characteristics
1	W1C	Sensitive to aromatic compounds
2	W5S	Highly sensitive to nitrogen oxides
3	W3C	Sensitive to ammonia and aromatic compounds
4	W6S	Selective toward hydrogen gas
5	W5C	Sensitive to alkanes and aromatic components
6	W1S	Sensitive to methane
7	W1W	Sensitive to sulfides
8	W2S	Sensitive to ethanol
9	W2W	Sensitive to aromatic compounds and organosulfur compounds
10	W3S	Sensitive to alkanes

### 2.4 GC-MS analysis conditions

#### 2.4.1 Sample pretreatment

The pre-prepared fresh solid samples were removed from the -80°C refrigerator, ground in liquid nitrogen, and vortexed to ensure uniform mixing. A 0.2 g aliquot of each sample was transferred to a tube, followed by the addition of 0.2 g sodium chloride powder and 20 μL internal standard solution(1,4-Di(methyl-d3)benzene-d4),and the concentration is 10 μg/mL (Fang et al., 2024). Extraction was then performed using automated headspace solid-phase microextraction (HS-SPME) for subsequent GC-MS analysis.

#### 2.4.2 Chromatographic conditions

A DB-5MS capillary column (30 m × 0.25 mm i.d., 0.25 μm film thickness) was employed for separation, with high-purity nitrogen (≥99.999%) as the carrier gas serving as the carrier gas at a flow rate of 1.2 mL/min. The inlet temperature was maintained at 250°C. The temperature program consisted of an initial hold at 40°C for 3.5 min, followed by a ramp to 100°C at 10°C/min, then to 180°C at 7°C/min, and finally to 280°C at 25°C/min, with a 5 min hold at the final temperature.

#### 2.4.3 Mass spectrometric conditions

An electron impact (EI) ion source was used with an ionization energy of 70 eV. The ion source temperature was 230°C, and the quadrupole temperature was 150°C. Selected ion monitoring (SIM) mode was adopted (GB 23200.8-2016).

### 2.5 Data analysis

Data preprocessing was first performed: R software was used for missing value imputation (with values set to 1/5 of the minimum observed value) and coefficient of variation (CV) filtering (CV < 0.5). Subsequently, qualitative and quantitative analysis of metabolites was conducted: qualitative identification was based on a self-built database (including retention time and characteristic ions), and the relative content was calculated using the internal standard semi-quantitative method. Multivariate statistical analyses including PCA and orthogonal partial least squares-discriminant analysis (OPLS-DA) were performed. The screening criteria for differential metabolites were |log_2_FC| ≥ 1 and p < 0.05. Finally, for functional annotation, metabolic pathway enrichment analysis was carried out using the KEGG database.KEGG enrichment analysis was performed using the R package clusterProfiler (version 4.1.0) with the hypergeometric test algorithm.The identified metabolites were annotated using the KEGG Compound Database (http://www.kegg.jp/kegg/compound/), and the annotated metabolites were subsequently mapped to the KEGG Pathway Database (http://www.kegg.jp/kegg/pathway.html). And data visualization supported by heatmaps and radar charts.

Prior to formal statistical testing, normality of seed residual weight data was verified using the Shapiro–Wilk test, and homogeneity of variances was examined simultaneously. All data satisfied the assumptions of normality and homogeneity of variances (P > 0.05). The core statistical method was two−way repeated−measures analysis of variance (ANOVA), supplemented by two−way ANOVA to identify highly significant differences (P < 0.0001). In this study, different metabolite treatments were set as the between−subjects factor, and sampling time points on days 3, 5, and 7 were included as the within−subjects repeated−measures factor. The analysis focused on evaluating the effects of metabolite treatments on pigeon feeding preference, as indicated by seed residual weight.

To avoid positional feeding bias in pigeons, the positions of control and treatment feed boxes within each cage were randomized daily. An incomplete Latin square design was applied to systematically account for two major confounding factors: (1) inherent between−cage variation in pigeon feeding behavior, and (2) daily fluctuations in environmental conditions (e.g., temperature, light intensity) that may influence avian feeding responses. This design ensured that each treatment was reliably tested across all experimental units.

### 2.6 Pigeon feeding validation experiment

Based on the preliminary metabolomic analysis, three metabolites were selected for investigation of their effects on feeding behavior and dose-response relationships in domesticated pigeons. The evaluated compounds included 2-methyl-1-butanethiol (purity ≥95%), furanone (purity ≥98%), and n-decanol (purity ≥98%), all verified by gas chromatography-mass spectrometry (GC-MS).

For stock solution preparation: each metabolite was individually dissolved in propylene glycol to prepare a 1% (v/v) stock solution. Storage conditions were optimized based on chemical stability, 2-methyl-1-butanethiol (light-sensitive): stored at -20°C in amber vials under dark conditions, Furanone and n-decanol: stored at 4°C.

For working solution dilution: prior to daily experiments, stock solutions were freshly diluted with distilled water to achieve test concentrations. The dose design for the three target metabolites adhered to three core principles: (1) concentrations were set below reported acute toxicity thresholds to avoid pigeon intoxication; (2) preliminary ranges were determined based on avian and mammalian behavioral studies of homologous compounds; (3) concentration effectiveness and safety were verified via body weight monitoring in preliminary pigeon trials.

2-methyl-1-butanethiol is a sulfur-containing volatile organic compound detectable by birds at ultra-low concentrations. Nevitt & Haberman reported that Leach’s storm-petrel (Oceanodroma leucorhoa) showed significant behavioral attraction to dimethyl sulfide (DMS, a structural analog) at a 100 μmol/L (≈0.001%) threshold (Nevitt and Haberman, 2003). Accordingly, sulfur-containing compounds within 0.001%–0.01% elicit observable avian behavioral responses. Despite limited acute toxicity data for thiols, the tested concentrations (0.005%, 50 ppm; 0.01%, 100 ppm) were far below acute intoxication levels based on analog extrapolation and safety guidelines. Preliminary 5-day exposure revealed no toxic symptoms and <5% body weight loss in pigeons.

Furanone is a natural fruit flavor agent widely used in the food industry. Per Sigma-Aldrich safety data (Sigma-Aldrich, 2023), its rat oral LD_50_ exceeds 1,400 mg/kg BW. For 400 g pigeons, maximum exposure via treated wheat intake (≈300 mg/kg BW) remained far below this LD_50_. No significant weight loss or abnormal behavior was observed after 5-day exposure.

N-decanol (1-decanol) is a long-chain aliphatic alcohol with a rat oral LD_50_ >5,000 mg/kg BW (Sigma-Aldrich, 2023). At 0.04% (400 ppm) and 0.08% (800 ppm), maximum pigeon exposure (≈300 mg/kg BW) accounted for only 6% of the LD_50_, demonstrating a wide safety margin. Five-day continuous exposure caused no marked weight loss (<5% initial BW) or abnormal behaviors. 2-methyl-1-butanethiol: 0.005% and 0.01% (v/v), Furanone: 0.04% and 0.08% (v/v) and n-decanol: 0.04% and 0.08% (v/v).For control Groups: the experiment included two control conditions. Vehicle control was distilled water, and solvent control was 1% (v/v) propylene glycol aqueous solution. These controls were implemented to account for potential solvent effects on pigeon behavior.

Prior to the behavioral experiment, clean, mature wheat grains of the cultivar Yongliang 4 was selected as the test substrate. Nine adult domesticated rock pigeons with similar body weights were randomly allocated to three groups and housed in three independent large cages (3 pigeons per cage). Prior to the experiment, all pigeons underwent an acclimatization period of at least 3 days and a time-limited feeding training of 8 hours per day to ensure stable feeding motivation during the test period. For each treatment, 40 g dry wheat grains were immersed in 100 mL of the corresponding test solution. The grains were soaked for 30 minutes with intermittent stirring gentle stirring (every 5 minutes) to ensure uniform exposure. After soaking, the grains were transferred to a stainless steel sieve and allowed to drain for 15 minutes to remove excess surface water. The initial moist grains weight was then measured using an analytical balance and recorded immediately before presentation to the pigeons. To control for potential confounding factors, an incomplete Latin square design was implemented to systematically balance both cage effects and daily temporal variations in experimental conditions.

In the first stage of the experiment, each cage was equipped with three feed boxes and one water box. The feed boxes contained mature Yongliang 4 seeds that had been pre-soaked for 30 minutes in one of the following solutions: distilled water (control), 1% (v/v) propylene glycol (solvent control), or a treatment solution containing one of the three metabolites (0.005% 2-methyl-1-butanethiol, 0.04% furanone, or 0.04% n-decanol). Daily feed consumption was calculated by weighing the remaining seeds after an 8-hour feeding period. and seed consumption data were recorded and analyzed after 3 and 5 days of feeding.

In the second stage of the experiment, a dose-response study with gradient concentration was conducted for each of the three metabolites, 2-methyl-1-butanethiol, furanone, and n-decanol. Each experimental cage (No. 1-3) was equipped with three feed boxes and one water box. The feed boxes were prepared as follows: for cage No. 1, control was water-treated Yongliang 4 grains, test concentrations were 0.005% and 0.01% (v/v) 2-methyl-1-butanethiol; for cage No. 2, control was water-treated Yongliang 4 grains, test concentrations were 0.04% and 0.08% (v/v) furanone; for cage No. 3, control was water-treated Yongliang 4 grains, test concentrations were 0.04% and 0.08% (v/v) n-decanol.

The grains were prepared using standardized protocols (30-minute soaking, 15-minute draining on a 2 mm sieve). Daily feed consumption was quantified by weighing residual grains after an 8-hour feeding period. The experiment spanned 7 days, with consumption data recorded at days 3, 5, and 7 post-treatment initiation for statistical analysis.

## 3 Results

### 3.1 Differential feeding preference of sparrows for waxy-ripe seeds from reciprocal crosses of Nongpin 5 and Mengjian 33

**Figure 1:**
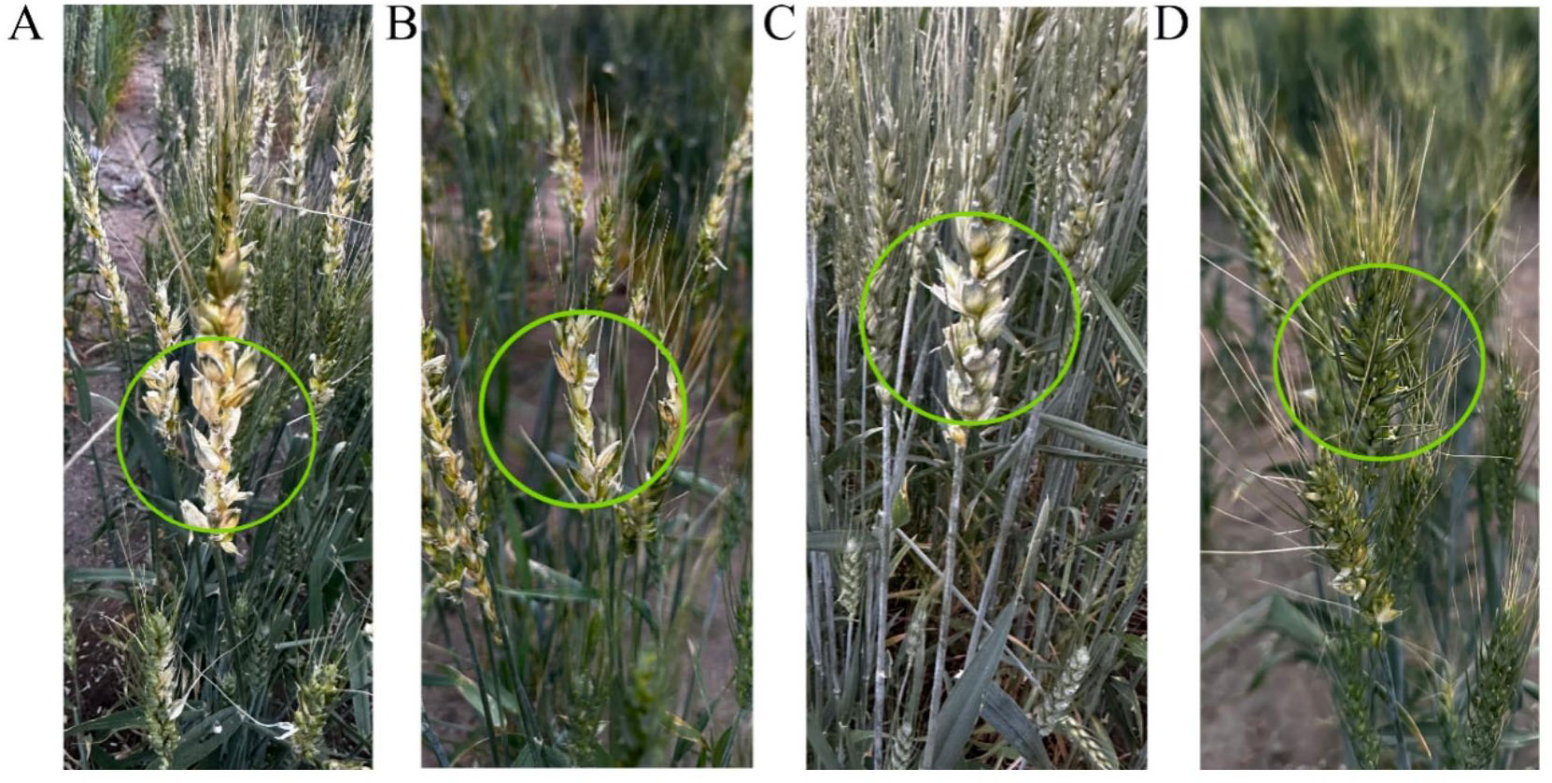
Observation of different wheat materials exhibiting distinct feeding preference during the wax-ripening stage. **(A)** Phenotypic observation of wax maturity in Nongpin 5; **(B)** Phenotypic observation of wax maturity in the F1 seeds from direct cross population (♀Nongpin 5×♂Mengjian 33); **(C)** Phenotypic observation of wax maturity in Mengjian 33; **(D)** Phenotypic observation of wax maturity in the F1 seeds from reciprocal cross population (♀Mengjian 33×♂Nongpin 5).

Field observations in wheat germplasm nurseries during the waxing stage, revealed a pronounced divergence in avian feeding behavior: house sparrows and wild rock pigens exhibited a significant feeding preference for the cultivar Nongpin 5 (**Figure 1A**), while demonstrating clear avoidance of Mengjian 33 (**Figure 1C**). To investigate the genetic basis of this behavioral disparity, we generated reciprocal crosses populations between Nongpin 5 and Mengjian 33 in 2024. During the waxing stage, field phenotypic surveys revealed that the direct cross population (with Nongpin 5 as the maternal parent) remained attractive to sparrows and wild rock pigeons (**Figure 1B**). In contrast, the reciprocal cross population (with Mengjian 33 as the maternal parent) was strongly rejected by house sparrows and wild rock pigens (**Figure 1D**). The above results indicated that the differential attractiveness of these two reciprocal crosses populations to birds is the same as the differential attractiveness of their maternal parents.

Furthermore, we present the grain loss rates caused by bird feeding quantified at the wheat waxy-ripening stage (35 days after pollination), corresponding to the day the photographs were captured. Statistical data show that the grain loss rate was 57.7% for Nongpin 5, 57.2% for F1seeds of ♀Nongpin 5 × ♂Mengjian 33, 9% for Mengjian 33, and 8.1% for F1seeds of ♀Mengjian 33 × ♂Nongpin 5.Grain loss rates among different wheat materials were compared using the chi−square test. No significant difference in grain loss rate was observed between Nongpin 5 and F_1_ grains from ♀Nongpin 5 × ♂Mengjian 33 (P > 0.05), nor between Mengjian 33 and F_1_ grains from ♀Mengjian 33 × ♂Nongpin 5 (P > 0.05). However, grain loss rates differed extremely significantly between the the group consisting of Nongpin 5 and its direct-cross F_1_ grains, and the group consisting of Mengjian 33 and its reciprocal-cross F_1_ grains (P < 0.01) (**Supplementary Table 1**).These marked differences in grain damage rates accurately reflect the significant disparities in bird feeding preferences across the four wheat materials.

### 3.2 Electronic nose analysis identified sulfides as key drivers of avian avoidance in F_1_seeds of reciprocal cross population

**Figure 2:**
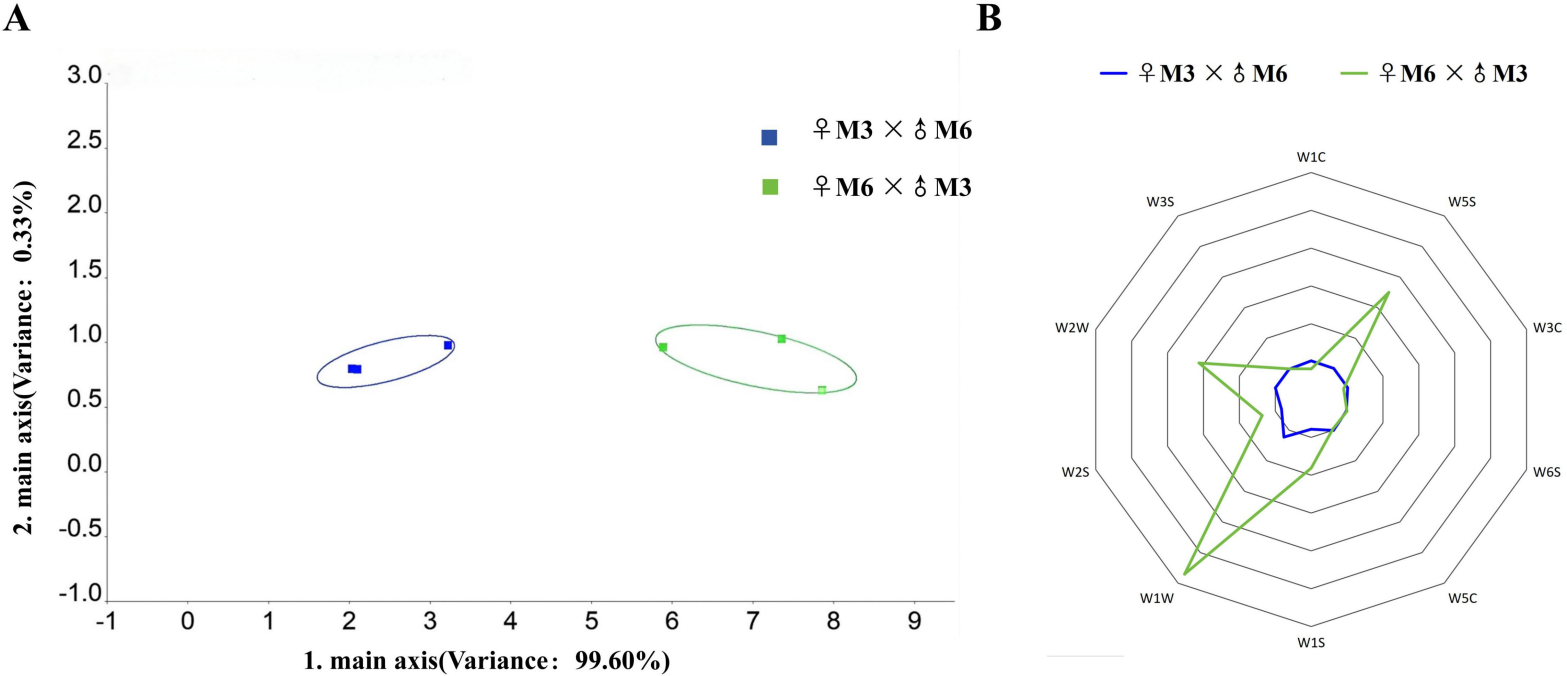
PCA analysis and response radar chart of electronic nose data. **(A)** PCA analysis of electronic nose response values; **(B)** Electronic Nose Radar Image; M3 and M6 presents the cultivar Nongmai 5 and Mengjian 33, respectively.

PCA of the electronic nose (E-nose) response values for wheat grains revealed that the cumulative variance contribution rate of principal components PC1 and PC2 reached 99.925% (99.598% for PC1 and 0.327% for PC2), significantly exceeding the 95% threshold (**Figure 2A**). This result indicates that these two principal components can fully capture the odor differences between the two sample groups and effectively substitute for all individual indicators in reflecting the odor variation characteristics of the samples.

E-nose analysis was conducted on the mixed samples from the direct cross population (bird-preferred type) and the reciprocal cross population (bird-avoided type), both at the waxy-ripe stage. Each mixed sample consisted of three biological replicates, resulting in a total of six samples analyzed. The E-nose data were visualized using a radar chart (**Figure 2B**). The radar chart revealed that sensor signals from the direct cross population samples were consistently weak, whereas three sensors in the reciprocal cross population samples exhibited significantly stronger responses: W5S (nitrogen oxides, response value 3.50), W1W (sulfides, 5.71), and W2W (aromatic components and organosulfur compounds, 3.13) in the reciprocal cross population samples were significantly stronger. This suggests that the reciprocal cross population samples contain higher concentrations of these volatile compounds, which are likely to be strongly perceived by avian olfactory systems. Notably, among the 10 sensors response values, the W1W sensor (a sulfide-specific sensor) showed the highest response in the reciprocal cross population samples, significantly exceeding other sensors. These findings collectively indicate that sulfide odors play a pivotal role in triggering avian avoidance behavior.

### 3.3 Combined analysis of key metabolities in reciprocal crosses populations based on values of |log_2_FC| and rOAV

**Figure 3:**
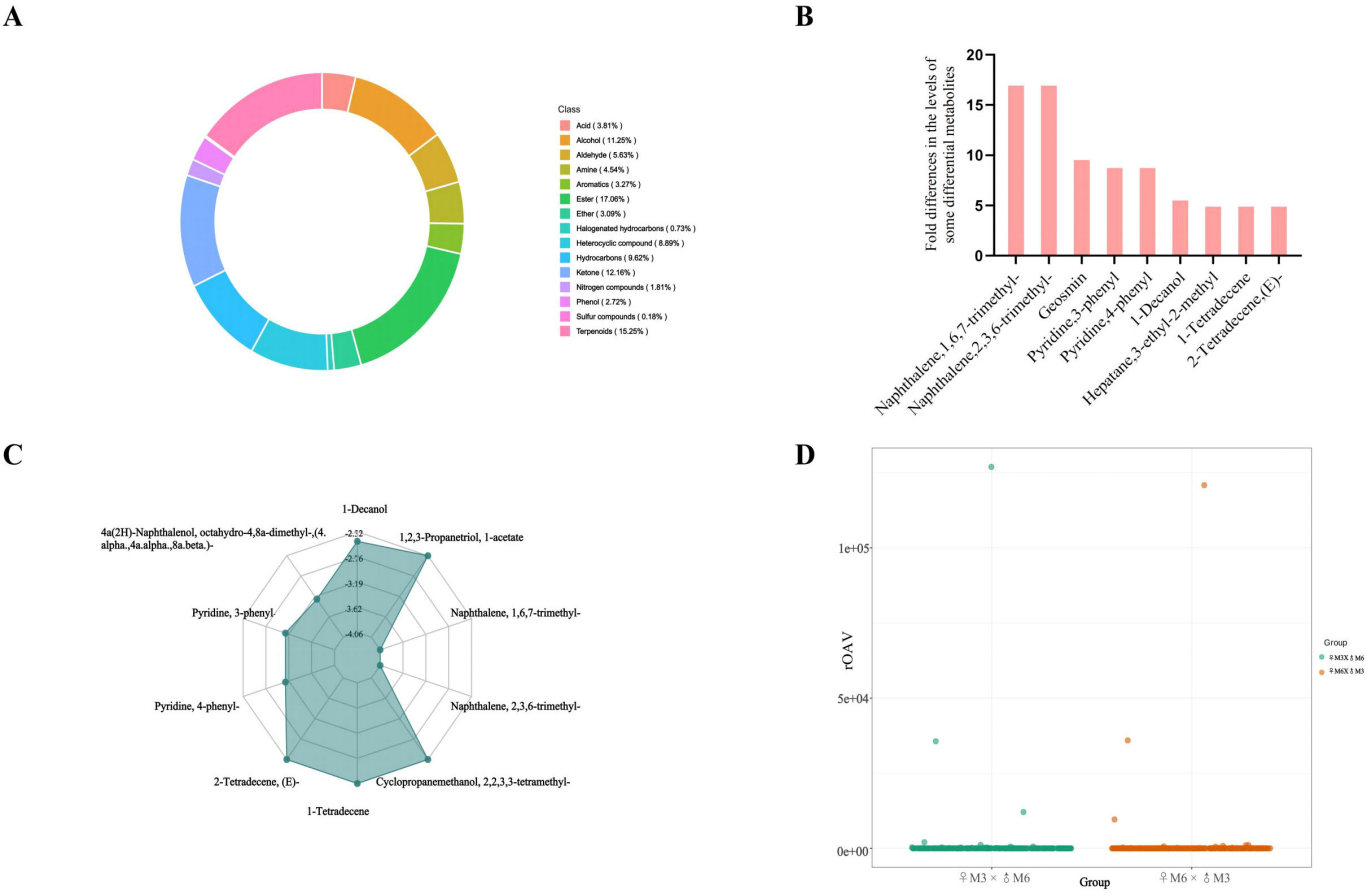
Differential metabolite distribution map and rOAV-based odor activity scatter plot. **(A)** Metabolite composition analysis chart; **(B)** Semi-quantitative fold change analysis of metabolite differences; **(C)** Radar chart of the top ten differential metabolites, ranked by absolute log_2_fold change values; **(D)** Scatter plot illustrating odor activity based on rOAV values.

Based on the gas chromatography-mass spectrometry (GC-MS) platform and the self-built database of Metware Biotechnology Co., Ltd., a total of 551 metabolites were identified, covering 16 chemical categories. Among these, esters (17.06%), terpenoids (15.25%), ketones (12.16%), and alcohols (11.25%) emerged as the dominant classes (**Figure 3A**). Notably, the direct cross population exhibited dramatically higher levels of specific metabolites, including trimethylnaphthalene and dimethyltetralol, with concentrations exceeding those in the reciprocal cross population by orders of magnitude (**Table 2**, **Figures 3B, C**). This stark contrast highlights profound differences in the metabolomic profiles between the two cross types. Furthermore, the direct and reciprocal cross populations displayed a distinct maternal bias in its metabolome composition. This observation suggests that the observed metabolite differentiation may be primarily governed by cytoplasmic genetic factors rather than nuclear genomic inheritance.

**Table 2:** Statistical analysis of relative abundance of selected metabolites.

Substance name	Primary classification	Molecular formula	M3×M6 content(μg/mL)	M6×M3 content(μg/mL)	Content difference multiple
1,6,7-Trimethylnaphthalene	Aromatic hydrocarbons	C13H14	0.0018	0.0001	17.2260
2,3,6-Trimethylnaphthalene	Aromatic hydrocarbons	C13H14	0.0018	0.0001	17.2260
Dimethylnaphthol	Alcohol	C12H22O	0.0061	0.0006	9.8271
3-Phenylpyridine	Heterocyclic Compounds	C11H9N	0.0052	0.0006	9.0216
4-Phenylpyridine	Heterocyclic Compounds	C11H9N	0.0052	0.0006	9.0216
1-Decanol	Alcohol	C10H22O	0.0021	0.0004	5.7981
3-Ethyl-2-methylheptane	Hydrocarbons	C10H22	0.0024	0.0005	5.1670
1-Tetradecene	Hydrocarbons	C14H28	0.0008	0.0001	5.1670
(E)-2-Tetradecen	Hydrocarbons	C14H28	0.0008	0.0001	5.1670

M3 and M6 present the cultivars Nongmai 5 and Mengjian 33, respectively.

The relative odor activity value (rOAV) is a core index for evaluating the contribution of flavor compounds to sample’s overall flavor profile. compounds are categorized as follows: those with rOAV ≥ 1 are defined as key flavor components; those with 0.1 ≤ rOAV < 1 play an important modifying role; and those with rOAV < 0.1 make no substantial contribution.

Based on the criterion of rOAV ≥ 1, 47 key flavor metabolites were screened out, Among these, aldehydes and alcohols each accounted for 21.28%, while ketones accounted for 17.02%. These three categories of compounds are hypothesized to be the core volatile substances regulating the flavor characteristics of wheat grains.

Combined with the rOAV scatter plot (**Figure 3D**), it can be observed that most of the data points for the direct cross population (green dots) were concentrated in the low rOAV value region. Similarly, the reciprocal cross population (orange dots) also exhibited three relatively high-value points. Further traceability analysis revealed that these six high-value points correspond to three core flavor compounds: 1-nonen-3-one, 2-methoxy-3,5-dimethylpyrazine, and (Z)-6-nonen-1-ol.

In terms of odor characteristics and biological functions: 1-nonen-3-one exhibits an extremely low odor threshold (0.1–1 ppb), enabling perception at trace concentrations. It is characterized by dual fruity and earthy notes, has been linked to lipid oxidation processes, and is classified as an off-flavor compound requiring stringent control in food applications (Xie et al., 2024). 2-Methoxy-3,5-dimethylpyrazine displays possesses a complex odor profile encompassing nutty, roasted, and moldy attributes. It is widely distributed across both food flavor systems and insect chemical communication pathways (Chatonnet et al., 2010; Quelal et al., 2023). (Z)-6-Nonen-1-ol imparts a fresh green odor with melon and cucumber nuances, featuring a low threshold and high aroma activity.a fresh green odor with melon and cucumber, featuring a low detection threshold and high aroma activity.

Regarding distribution differences between the two cross populations: the direct cross population (preferred by birds) showed relatively higher contents of 1-nonen-3-one and (Z)-6-nonen-1-ol, whereas whereas the reciprocal cross population (avoided by birds) exhibited greater abundance of 2-methoxy-3,5-dimethylpyrazine. Although the fold changes of these compounds between the two groups were not statistically significant, all three are aromatic odorants whose sensory profiles align well with E-nose detection data. Notably, the reciprocal cross population generated strong response signals (e.g., W5S, W1W sensors), which may be directly attributed to the high content and potent aromatic activity of 2-methoxy-3,5-dimethylpyrazine. Based on these comprehensive analysis, these three core aromatic compounds likely influence birds’ foraging strategy selection through their distinct aromatic activities.

Compared with the direct cross population, the key up-regulated metabolites in the reciprocal cross population, screened based on the criteria of top six-fold changes (|log_2_FC|) and rOAV > 1, were 2-methyl-1-butanethiol and 4-hydroxy-2,5-dimethyl-3(2H)-furanone (**Table 3**). In addition, the significantly higher signals on the W1W (sulfide) sensors were exhibited in reciprocal cross population. These results suggests that metabolites such as 2-methyl-1-butanethiol possess “warning odor” characteristics and trigger strong negative stimulus in the avian olfactory system, resulting in obvious feeding avoidance behavior by birds towards the reciprocal cross population. Conversely, based on the criteria of top 15-fold changes (|log_2_FC|) and rOAV > 1, the key down-regulated metabolites in the reciprocal cross population were dimethyltetralol and (E)-2-dodecenal (**Table 3**). It is hypothesized that the smoky aroma of dimethyltetralol and the citrus-like odor of compounds such as (E)-2-dodecenal are highly analogous to the volatile components emitted by various types of ripe fruits, which consequently enhances the foraging preference of avian species.

**Table 3:** Key metabolite concentrations and rOAV values.

Substance name	M3×M6 content (μg/mL)	M6×M3 content (μg/mL)	Difference multiple (|log2FC|)	Up/down regulation	rOAV value for M3×M6 group	rOAV value for M6×M3 group
2-Methyl-1-butanethiol	0.02602	0.08142	3.233	Up	260.24840	814.19795
4-Hydroxy-2,5-dimethyl-3(2H)-furanone	0.00519	0.01496	2.979	Up	5.19061	14.96411
Dimethylnaphthol	0.00610	0.00062	9.829	Down	29.06763	2.95791
(E)-2-Dodecenal	0.01034	0.00200	5.167	Down	1.41660	0.27416

M3 and M6 present the cultivars Nongmai 5 and Mengjian 33, respectively.

### 3.5 A Metabolic network of dimethyltetralol, hordenine, and l-pipecolic acid mediates wheat grains responses to avian foraging choices

**Figure 4:**
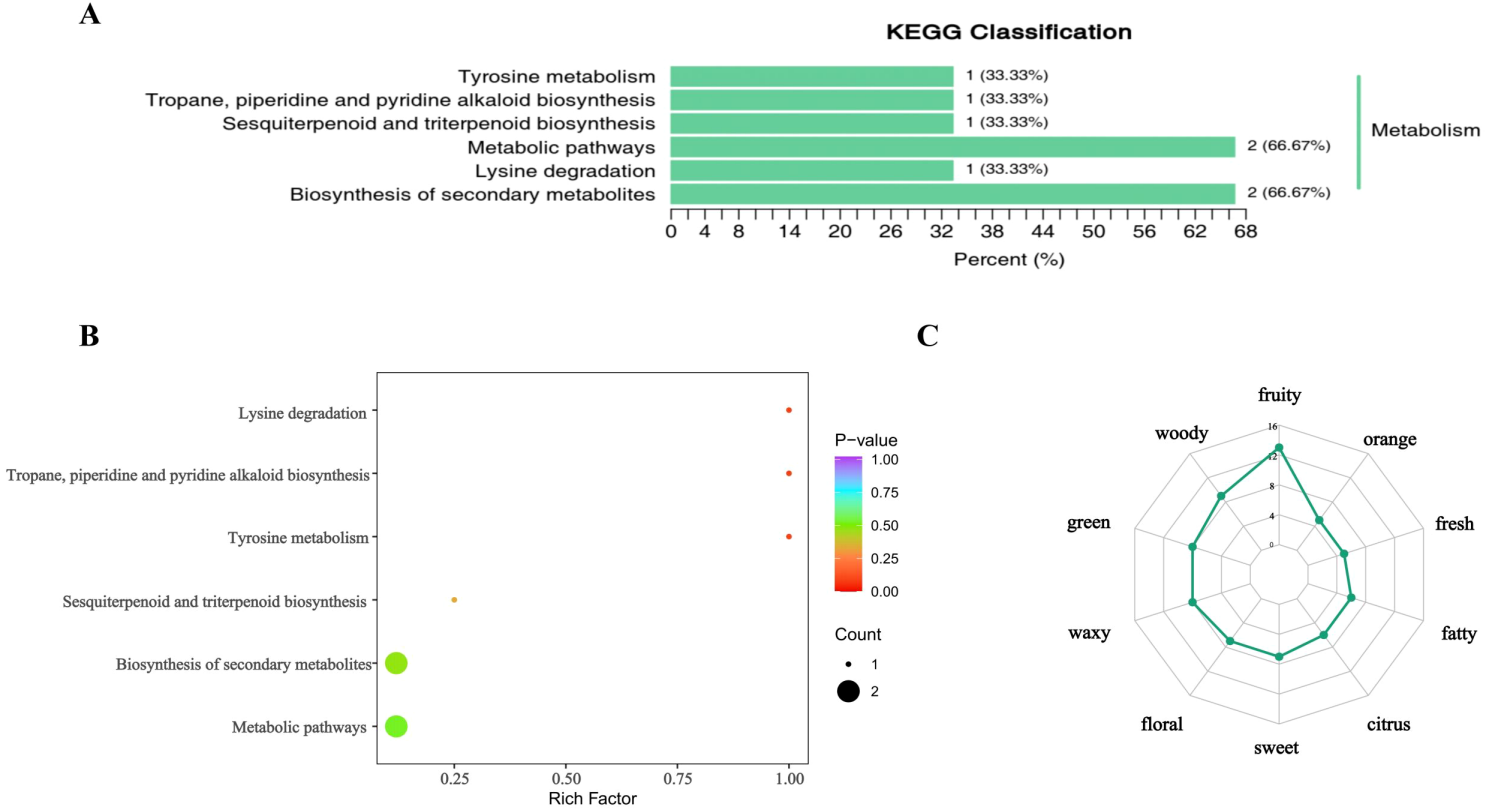
KEGG enrichment analysis and flavor sensory analysis diagram. **(A)** Classification of differential metabolite pathways; **(B)** Enrichment map of differential metabolite pathways; **(C)** Radar image of sensory flavor characteristics analysis of differential metabolites.

The KEGG pathway classification revealed distinct patterns in metabolism-related pathways (**Figure 4A**). Specifically, four pathways of tyrosine metabolism, tropane/piperidine/pyridine alkaloid biosynthesis, sesquiterpene and triterpene biosynthesi, and lysine degradation, each involved one type of related substance, collectively accounting for 33.33% of the total. In contrast, two pathways of metabolic pathways and secondary metabolite biosynthesis pathway, involved two type of substances each, contributing 66.67% of the total (**Table 4**, **Figure 4A**). Among the three DEMs, dimethyltetralol and hordenine were identified as down-regulated, while L-pipecolic acid exhibited up-regulated (**Figure 4B**).

**Table 4:** Annotated metabolic pathways of DEMs.

Substance name	Primary classification	M6×M3 content(μg/mL)	M3×M6 content(μg/mL)	Fold_change	Type	KEGG map
Dimethylnaphthol	Alcohol	6336.1797	60253.7952	0.1052	down	ko00909,ko01110
Hordenine	Phenol	8385.1753	37274.2998	0.2250	down	ko00350,ko01100
L-piperidinecarboxylic acid	acid	11768.0907	5181.8178	2.2710	up	ko00310,ko00960, ko01100,ko01110

M3 and M6 present the cultivars Nongmai 5 and Mengjian 33, respectively.

Compared with the direct cross population, dimethyltetrahydronaphthol and hordenine were identified as down-regulated metabolites in the reciprocal cross population, which are speculated to affect avian foraging behavior via their aromatic odor. In contrast, L-pipecolic acid, as an up-regulated metabolite, exhibits a highly volatile defensive odor that aligns well with the strong response of the reciprocal cross population to W2W (aromatic) odors in the electronic nose data. It is likely that its unpleasant odor is perceived by birds, leading to feeding avoidance behavior. This substance is also speculated to play a role in wheat’s stress resistance and defense mechanisms. In summary, dimethyltetrahydronaphthol, hordenine, and L-pipecolic acid constitute a critical set of metabolites that cooperatively regulate wheat’s response to avian foraging choices by playing distinct roles in defense enhancement, attraction, and deterrence. This provides a foundational metabolic basis for elucidating the chemical communication mechanism between wheat grains and birds.

### 3.6 Sensory flavor analysis reveals: woody-lipid-fruity aroma synergy attracts avian foraging, while green aroma deters

To intuitively visualize the sensory flavor profiles of DEMs, we selected the top 10 flavor indices with the highest annotation counts and generated a radar chart (**Figure 4C**). The intensity of the dominant flavor characteristics exhibited by these DEMs was ranked from strong to weak as follows: Strongest: fruity, citrus and sweet; Medium: floral, fresh*, fatty and waxy; Weakest: green, woody and orange.

Compared with the direct cross population, the up-regulated DEMs in the reciprocal cross population include several annotated compounds (for example (Z)-3,7-dimethyl-2,6-octadien-1-yl propionate, 1,1-diethoxyheptane and 4-hydroxy-2,5-dimethyl-3(2H)-furanone) with distinct sensory profiles. (Z)-3,7-dimethyl-2,6-octadien-1-yl propionate exhibits green and floral aromas; 1,1-diethoxyheptane presents complex flavors encompassing green and waxy; 4-hydroxy-2,5-dimethyl-3(2H)-furanone is characterized by a dominant sweet aroma. Notably, the green aroma compounds constitute a higher proportion in bird-avoiding group (reciprocal cross population). In natural environments, green aroma is frequently associated with plant immaturity. It is hypothesized that these volatile signals may convey cues of immaturity or low nutritional value to avian sensory systems, thereby effectively reducing birds’ foraging willingness. In addition, the down-regulated DEMs in the reciprocal cross population exhibit dominant flavors profiles of fruity, citrus, woody and lipid notes, For example, 1-decanol and (E)-2-dodecenal carry a citrus aroma; dimethyltetralol contributes a woody aroma. This combination of flavors closely mimics the odors profiles of mature fruits or high-lipid foods. Mature fruits typically emit fruity aroma as a hallmark of ripeness, while the presence of woody and lipid notes further enhances the simulation of high-lipid foods or mature fruits characteristics. E-nose data corroborate these findings, showing that these flavor profiles correspond to elevated response values for aromatic compounds. Such signals are highly sensitive to avian detection, likely promoting birds’ foraging preference for wheat grains with these specific flavor characteristics.

In summary, it is speculated that wheat cultivars preferred by birds construct an attractive odor profile characterized by high levels of woody, lipid, and citrus-like aromatic volatiles. This profile not only directly stimulates birds’ foraging desire but also signals high grain maturity and rich energy content, thereby exerting a positive guiding effect on their foraging behavior. In contrast, green aromas may transmit signals of fruit immaturity, thereby exerting a negative avoidance effect on bird foraging.

### 3.7 Dimethyl-1-butanethiol repels domesticated pigeons whereas n-decanol attracts

**Figure 5:**
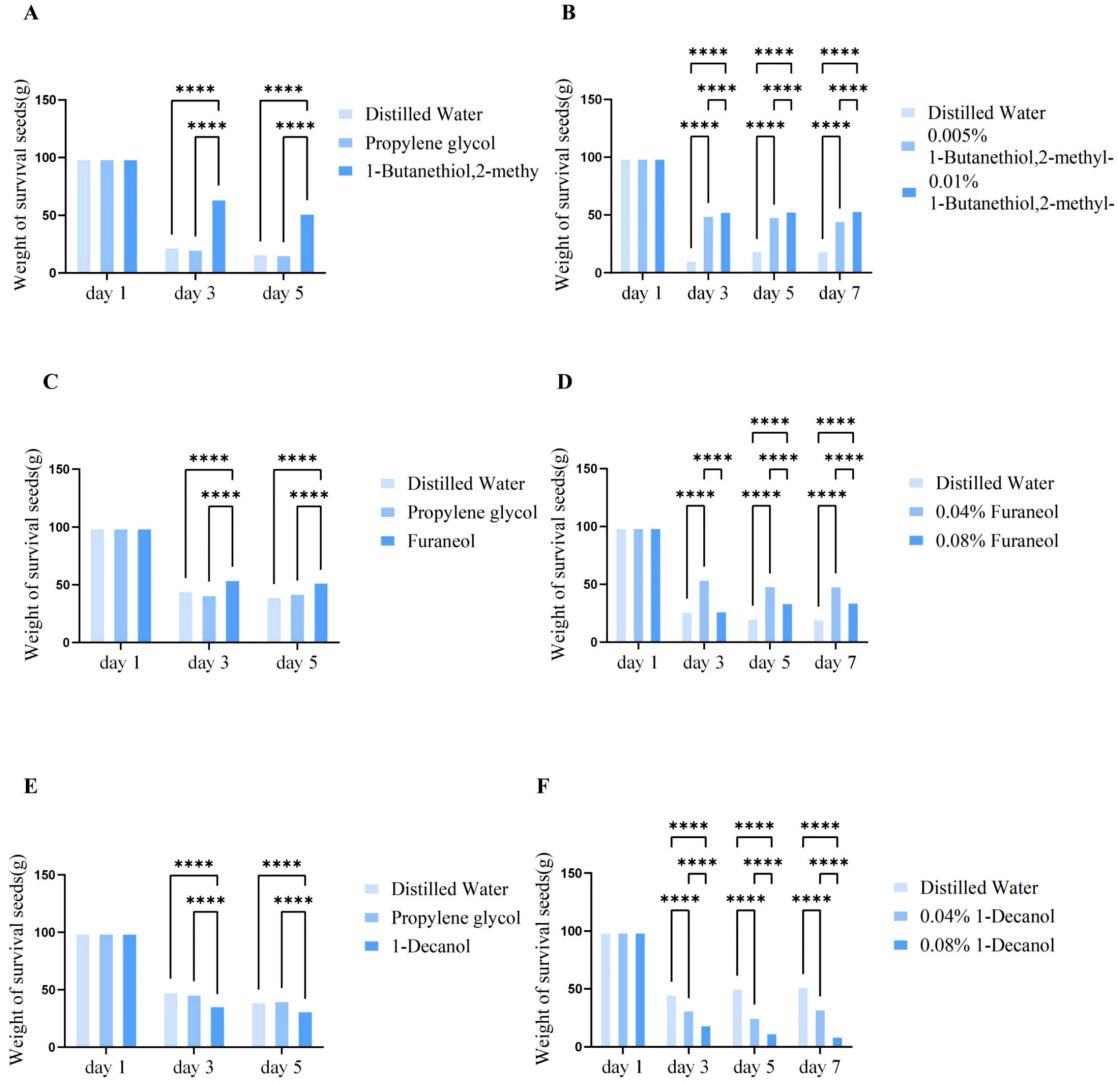
Residual wheat grains after treatment with different metabolites. **(A, B)** Residual wheat grain levels after treatment with different concentrations of dimethyl-1-butanethiol; **(C, D)** Residual wheat grain levels after treatment with different concentrations of furanone; **(E, F)** Residual wheat grain levels after treatment with different concentrations of n-decanol. **(A-F)** Differences were analyzed by two-way ANOVA.**** P < 0.001.

In the experiment where wheat grains treated with 0.005% Dimethyl-1-butanethiol were offered to pigeons, the residual wheat grains weight was quantified at 3 and 5 days post-feeding. The results revealed that the residual amount of wheat grains soaked in 0.005% dimethyl-1-butanethiol was significant higher than that of water-treated or 1% propylene glycol-treated grains (*P* < 0.001). In contrast, no significant difference in the residual wheat grains weight was observed between the water control group and the propylene glycol control group (*P* > 0.05) (**Figure 5A**; **Supplementary Video S1**). These findings indicated that propylene glycol, as the solvent for the target metabolites Dimethyl-1-butanethiol, furanone and n-decanol, exerted no significant interference on the feeding behavior of domesticated pigeons, thereby eliminating its potential confounding effect on the experimental outcomes. Furthermore, dimethyl-1-butanethiol significantly reduced pigeons’ feeding preference for wheat grains.

In another experiment, wheat grains treated with 0.005% or 0.01% dimethyl-1-butanethiol were offered to pigeons, and residual grain weight was measured at 3, 5, and 7 days post-feeding. The results revealed that the residual weight of water-treated grains was significantly lower than those treated with either 0.005% or 0.01% dimethyl-1-butanethiol. Furthermore, the residual weight of grains treated with 0.005% dimethyl-1-butanethiol was significantly lower than that of the 0.01% treatment group. Statistically significant differences were observed among all three groups (*P* < 0.001) (**Figure 5B**; **Supplementary Video S2**). Collectively, these findings suggest that pigeons’ feeding interest in grains decreased with increasing concentrations of dimethyl-1-butanethiol increases.

In an experiment where wheat grain treated with 0.04% or 0.08% furanone were offered to pigeons, the residual grain weight was measured at 3 and 5 days post-feeding. The results reaveled a consistent trend in residual weight across both time points: grains treated with 0.04% furanone>water-treat control>0.08% furanone-treated grains (**Figures 5C, D**; **Supplementary Videos S3**, **S4**). Collectively, these feeding experiments demonstrated that the inhibitory effect of pigeons’ wheat consumption preference did not intensify with increasing furanone concentration. This unexpected pattern suggests that furanone’s regulatory effect on pigeon feeding behavior may not be mediated by a dose-dependent mechanism.

In another experiment, wheat grains treated with 0.04% or 0.08% n-decanol were offered to pigeons. Residual wheat grains weight was measured at 3, 5, and 7 days post-feeding (**Figures 5E, F**; **Supplementary Videos 5**, **6**). The results showed that the residual weight of water-treated grains was significantly higher than that of grains treated with either 0.04% or 0.08% n-decanol. Furthermore, the residual weight in the 0.08% n-decanol treatment group was significantly lower than that in the 0.04% treatment group. Collectively, these feeding experiments demonstrated that the residual grains weight decreaced in a concentration-dependent manner with increasing n-decanol concentration. In other words, pigeons’ feeding preference for wheat was significantly enhanced as the concentration of n-decanol increased.

## 4 Conclusions

In this study, electronic nose (E-nose) technology combined with untargeted metabolomics was employed to systematically characterize the key differentially expressed metabolites (DEMs) between bird-preferred and bird-avoided wheat grains. The foraging preferences mediated by these metabolites were further validated through pigeon behavioral assays. The main conclusions are summarized as follows.

### 4.1 Aroma composition contributes to bird preference for wheat grains

Bird-preferred wheat grains are characterized by elevated levels of aromatic volatile components. Flavor sensory analysis demonstrates that these grains exhibit a strong aroma profile dominated by woody, lipid, and fruity notes. This specific combination of volatile aromas is proposed to act as a key attractant underlying bird foraging preference.

### 4.2 Organosulfur compounds are responsible for bird avoidance

Bird-avoided wheat grains are rich in sulfides and aromatic organosulfur compounds. Combined analysis of metabolomic and E-nose data indicates that 2-methyl-1-butanethiol is a key metabolite responsible for inducing bird avoidance behavior. This compound may serve as a critical repellent signal for birds.

### 4.3 Key metabolites directly regulate bird feeding behavior

**Figure 6:**
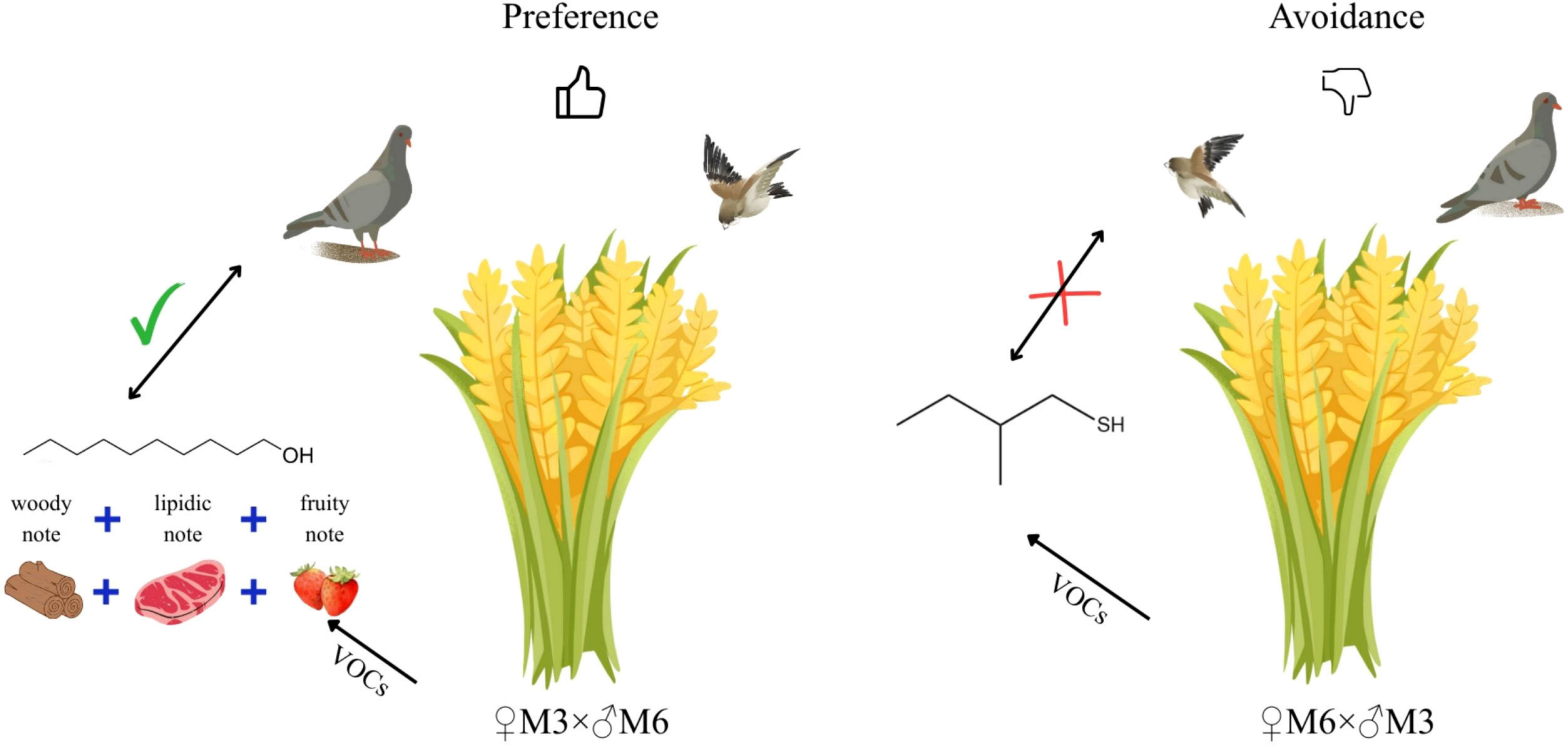
Summary diagram of conclusions.

Behavioral assays confirm the distinct roles of the two identified metabolites in modulating bird feeding choices. Further feeding experiments show that domestic pigeons exhibit a significant preference for wheat grains treated with n-decanol, while actively avoiding grains treated with 2-methyl-1-butanethiol. These results verify that the two metabolites can independently trigger attractive or aversive responses in birds, respectively, and play decisive roles in regulating bird foraging selectivity toward wheat grains (**Figure 6**).

## 5 Discussion

### 5.1 Efficiency of rOAV based screening for identifying key flavor metabolites in non targeted metabolomics

To accurately identify key differential metabolites in the untargeted metabolomic analysis between the two sample groups, we performed a screening strategy based on metabolite content fold change (log_2_FC) and rOAV. This approach is supported by extensive research demonstrating that metabolites selected through these criteria not only exhibit statistically significant content differences but also likely play a pivotal role in shaping the overall odor profile of samples. For instance, recent studies have leveraged rOAV values to analyze aroma component disparities in Keemun black tea and investigate flavor variations induced by lactic acid bacteria and yeast fermentation (Huang et al., 2022). These findings provide critical insights for enhancing flavor profiles and optimizing product quality in insect-derived food systems (Cha et al., 2024). Moreover, the rOAV-based screening in this study successfully identified key flavor metabolites (**Table 3**), which were identified closely related to bird feeding behavior (**Figure 5**).

### 5.2 The content of VOCs in wheat grains is also a major factor affecting bird feeding preferences

Combined analysis of metabolomics and E-nose data revealed that the core attractive flavor profile of bird-preferred wheat is characterized by “fruity-woody-citrusy aromas” aromatic notes. Among these, dinaphthalenol, (E)-2-dodecenal, hordenine, n-decanol and Phenylacetaldehyde were identified as key functional metabolites.Volatile organic compounds (VOCs) with such sensory characteristics are evolutionarily conserved signals for avian foraging, as they are closely associated with the ripeness and nutritional richness of plant reproductive organs, and plants have evolved to secrete specific VOC blends to mediate interactions with seed-dispersing birds (Bergman et al., 2025). Furthermore, Dimethyltetralol is localized in the biosynthesis pathways of sesquiterpenoids and triterpenoids as well as secondary metabolites, having the character of insect-repellent and antibacterial activities (Piesik et al., 2012), suggesting that its elevated abundance in bird-preferred wheat grains may be directly linked to the crop’s enhanced defense mechanisms against biotic stressors while simultaneously serving as an olfactory attractant for birds. Phenylacetaldehyde possesses a sweet aroma and has been shown to attract birds such as hummingbirds for feeding (Kessler et al., 2008), and its methylated products (e.g., N-methylphenethylamine) may play a regulatory role in avian feeding behavior by modulating olfactory perception (Zhang and Jander, 2022). Notably, the dominance of woody-lipid-fruity VOCs in bird-preferred wheat aligns with the general pattern of plant VOC evolution for avian mutualism, where non-toxic, nutrient-associated odor signals are prioritized to attract seed-dispersing avian species (Bergman et al., 2025).

For avian foraging decision-making in agricultural habitats, the relative importance of olfactory and visual cues is context-dependent (Rubene et al., 2018). In the wheat germplasm nursery of this study, the uniform planting environment minimized the variation in visual cues (e.g., plant height, grain color) between the reciprocal cross populations and parental cultivars, making olfactory cues the primary sensory basis for house sparrows and wild rock pigeons to distinguish wheat grains. This finding is consistent with field experiments on farmland birds, which demonstrated that birds prioritize olfactory cues (e.g., herbivore-induced plant volatiles, HIPVs) for foraging when visual information is consistent, and the olfactory priority decreases only when visual cues show significant variation (Rubene et al., 2018). The high abundance of attractive VOCs such as n-decanol in the direct cross population thus becomes a dominant olfactory signal, guiding avian foraging preference in the homogeneous agricultural environment.

The core feeding-deterrent factor of bird-avoided wheat comprises sulfides, primarily 2-methyl-1-butanethiol, supplemented by L-pipecolic acid and 4-hydroxy-2,5-dimethyl-3(2H)-furanone (HDMF). This composition aligns with the strong response observed on the sulfide sensor of the electronic nose (E-nose), confirming the relevance of these compounds in avian feeding aversion. Avian species exhibit extremely high olfactory sensitivity to sulfur-containing compounds, with many seabirds capable of detecting dimethyl sulfide (DMS) at the nanogram level, and sulfur compounds serving as core foraging or avoidance cues in avian olfactory systems (Dell’ariccia et al., 2014).As a thiol compound with high odor activity, 2-methyl-1-butanethiol exhibits sensory characteristics of sulfur-like, rotten onion, and skunk-like unpleasant odors, which are perceptible even at trace concentrations (Devos, 1990). Thiol compounds such as hydrogen sulfide have been proven to trigger significant avoidance behavior in gallinaceous birds such as domestic chickens, and this repellent effect is a conserved behavioral response of avian species to harmful or spoiled organic matter-associated olfactory signals (Mckeegan et al., 2005). In beer and wine production, 2-methyl-1-butanethiol is a key off-flavor contributor linked to lightstruck odor and reductive sulfur off-odors (Gros and Collin, 2012), and its accumulation in the reciprocal cross population of wheat grains thus acts as a “warning odor” that activates the innate avoidance mechanism of birds.

HDMF demonstrates a paradoxical role: while it inherently possesses caramel and strawberry aroma and is widely utilized as a flavoring agent in the food industry, its concentration is significantly elevated in bird-avoided wheat grains. Behavioral assays reveal that low concentration of HDMF (0.04%) furanone reduce avian feeding preference, whereas the inhibitory effect diminishes at a high concentration (0.08%). This study suggests that in the avian olfactory system, negative sensory cues (such as the rotten odor of sulfides) are assigned greater behavioral weight than positive signals. Furthermore, the feeding-deterrent effect of HDMF demonstrates a concentration-dependent threshold. The mechanism behind this dose-response pattern requires further investigation.

### 5.3 VOCs in wheat grains associated with avian feeding preference determined by extranuclear genes or maternal influences

From a genetic perspective, significant differences of bird feeding preference were observed in F1 seeds derived from direct and reciprocal crosses populations and the metabolomic profiles of the two populations showed strong differentiation: the direct cross population is enriched in aromatic components and attractive metabolites such as dinaphthalenol and n-decanol, while the reciprocal cross population is enriched in repellent components such as sulfides including 2-methyl-1-butanethiol, with the maximum fold difference in the content of key metabolites exceeding 17 times. These phenotypic disparities do not conform to the principle of nuclear gene Mendelian inheritance.and the most typical genetic characteristic of such reciprocal cross phenotypic inconsistency is maternal effect or cytoplasmic inheritance (Roach and Wulff, 2009). The cytoplasmic genome of angiosperms, including plastid and mitochondrial genomes, is strictly maternally transmitted, and the paternal organelles are cleared during fertilization, which is the molecular basis for the metabolic and phenotypic differences of reciprocal cross progeny (Chung, 2025). Crop cytoplasmic genomes and maternal genetic effects directly regulate the synthesis of secondary metabolites such as aromatic compounds and sulfides, and are key determinants of crop quality and phenotypes related to herbivore foraging (Chu et al., 2025). So,The core intrinsic mechanism underlying these feeding behavior-associated differences is hypothesized to be closely linked to extranuclear inheritance (cytoplasmic inheritance) or maternal influence, as evidenced by the inconsistent phenotypes observed in these reciprocal crosses populations—a hallmark characteristic of cytoplasmic or maternal effects. Further speculation suggests that F_1_ seeds, Nongpin 5 serveing as the female parent, may carry key genes regulating the synthesis of aromatic metabolites or activate the biosynthesis pathway of attractive flavor substances through nuclear-cytoplasmic or maternal influence interactions. In contrast, F_1_ seeds, Mengjian 33 as the female parent, may carry key genes promoting the accumulation of repellent metabolites such as sulfides and aromatic organosulfur compounds, ultimately driving the divergent feeding preferences observed between the direct and reciprocal cross populations. So, this research opens a new avenue for crop breeding, enabling solutions to bird damage while advancing flavor-oriented breeding practices.

### 5.4 Strategies for designing wheat breeding for tailoring flavor profiles and mitigate avian pest pressure

This study not only elucidates the chemical mechanism of wheat grain VOCs mediating avian foraging preference but also opens a new avenue for crop breeding. The identification of key attractive/repellent metabolites and their genetic regulation basis provides a theoretical foundation for the molecular design breeding of wheat—by editing cytoplasmic or nuclear-cytoplasmic interaction genes to modulate the synthesis of flavor-related metabolites, wheat cultivars with intrinsic bird repellency can be developed, which is an eco-friendly strategy to mitigate avian pest pressure in wheat production. Future research can focus on cloning the key cytoplasmic genes regulating the synthesis of sulfides and aromatic compounds in wheat, and verifying their functions through transgenic and gene editing technologies; meanwhile, field trials of the bred bird-repellent wheat cultivars should be conducted to evaluate their actual effect on reducing avian damage and their agronomic traits, to realize the practical application of this research result in agricultural production. In addition, exploring the combined effect of olfactory and visual cues on avian foraging behavior of wheat in complex agricultural ecosystems can further improve the theoretical system of plant-avian chemical communication and provide more comprehensive strategies for avian pest management.

## Data Availability

The data analyzed in this study is subject to the following licenses/restrictions: No raw data submission is required for the widely targeted metabolomics. Requests to access these datasets should be directed to 707299001@qq.com.

## Supplementary material

Supplementary Video 1 First-stage feeding of 1-dimethylbutanethiol.

Supplementary Video 2 Second-stage feeding of 1-dimethylbutanethio.

Supplementary Video 3 First-stage feeding of furanone.

Supplementary Video 4 Second-stage feeding of furanone.

Supplementary Video 5 First-stage feeding of n-decanol.

Supplementary Video 6 Second-stage feeding of n-decanol.

Supplementary Table 1 Grain loss rate of wheat at the waxy ripe stage.
