# Supplementary material for: Mechanism of wax-ripening wheat seeds attracting birds based on GC-MS widely targeted metabolomics and electronic nose technology
Source: Front Plant Sci. 2026 Apr 24;17:1805496. doi: 10.3389/fpls.2026.1805496 (PMC13153126; doi:10.3389/fpls.2026.1805496)
Supplement: Supplementary Table 1 — Grain loss rate of wheat at the waxy ripe stage. [file Table1.pdf]

Supplemental Table 1 Grain loss rate of wheat at the waxy ripe stage

Variety Name	Expected Number of F_1 Grains	Actual Number of F_1 Grains Harvested	Loss Rate（%）
Nongpin 5	950	402	57.7%
♀Nongpin 5 × ♂Mengjian 33	407	175	57.0%
Mengjian 33	977	889	9.0%
♀Mengjian 33 × ♂Nongpin 5	421	388	7.8%
